# Supplementary material for: Identification of Freezing-Responsive microRNAs and Their Targets in Chinese Jujube by Small RNA and Degradome Sequencing
Source: Int J Mol Sci. 2026 Sep 4;27(17):7912. doi: 10.3390/ijms27177912 (PMC13566890; doi:10.3390/ijms27177912)
Supplement: Supplementary file 1 [file ijms-27-07912-s001.zip › Supplementary Material S2/novel_miR_63.pdf]

Provisional ID	:	novel_miR_63
Score total	:	7166.8
Score for star read(s)	:	3.9
Score for read counts	:	7158.1
Score for mfe	:	3.2
Score for randfold	:	1.6
Score for cons. seed	:	
Total read count	:	14052
Mature read count	:	9107
Loop read count	:	29
Star read count	:	4916

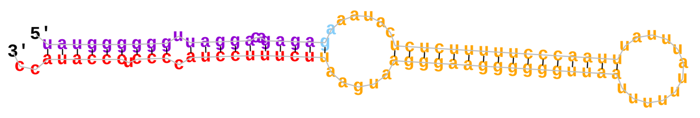

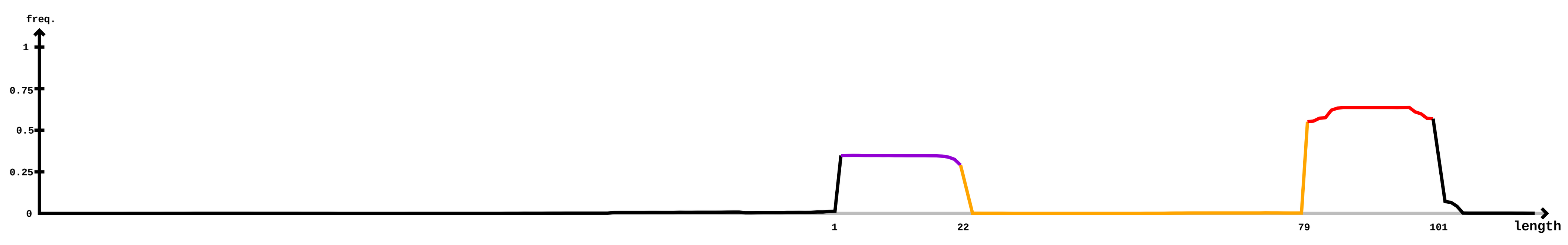

Star   Mature

```
5'- cuuuucuuuuuuucaccaaaaucuagacucaauuaauuucucuuuucccuuccuccaccaucugguguauuucuuuucucacuaaaaggauggaagagggaaaggcuuaagagugugggugguggaagugauagguaugggggguuaggacagagagaaauacucucuuuuucccaauuuauuuauuuuuuaauuggggggaagggaaugaauucuuuccuaccccucccauaccauucauuuccauccuuuc -3'  obs
    cuuuucuuuuuuucaccaaaaucuagacucaauuaauuucucuuuucccuuccuccaccaucugguguauuucuuuucucacuaaaaggauggaagagggaaaggcuuaagagugugggugguggaagugauagguaugggggguuaggacagagagaaauacucucuuuuucccaauuuauuuauuuuuuaauuggggggaagggaaugaauucuuuccuaccccucccauaccauucauuuccauccuuuc       exp
    ......................(.((((.....((((((((((((..((((.((((....))))..................)))).)))))))))))))......)))).)(((((((((((.(((((..(((((......(((((((((((((((............))))))))))))))).....)))))))))).))).))))))))..))))))))))))....
```

read	reads	mm	sample
caccaaaaucuagacucaauu	1	0	LT-1
ccaaaaucuagacucaauuaauuucucu	1	0	JCK-2
aaucuagacucaauuaauuucuc	1	0	LT-3
uagacucaauuaauuucucuuuuc	1	0	JCK-1
agacucaauuaauuucucuuuucc	1	0	JCK-1
agacucaauuaauuucucuuuucc	1	0	JCK-2
agacucaauuaauuucucuuuucc	1	0	JCK-3
ucugguguauuucuuuucuc	2	0	JCK-3
ucucacuaaaaggauggaagagggaa	2	0	LT-1
ucucacuaaaaggauggaagagggaaagg	1	0	JCK-1
ucucacuaaaaggauggaagagggaaagg	1	0	JCK-3
cucacuaaaaggauggaagagggaaagg	1	0	JCK-3
cacuaaaaggauggaagagg	1	0	LT-3
cacuaaaaggauggaagagggaaagg	1	0	JCK-2
cacuaaaaggauggaagagggaaaggA	2	1	JCK-3
cacuaaaaggauggaagagggaaaggA	2	1	JCK-2
aaaaggauggaagagggaaagg	4	0	LCK-3
aaaaggauggaagagggaaagg	1	0	LT-3
aaaggauggaagagggaaagg	1	0	LT-3
aggauggaagagggaaagg	3	0	LT-2
agagggaaaggcuuaagagu	5	0	LT-2
agagggaaaggcuuaagagug	1	0	LT-3
agagggaaaggcuuaagagugu	36	0	LCK-2
agagggaaaggcuuaagagugu	21	0	LCK-1
gagggaaaggcuuaagagug	1	0	JCK-2
Cgggaaaggcuuaagagugugggg	1	1	LT-1
aaaggcuuaagagugugggugg	1	0	LT-3
aaaggcuuaagagugugggugguggaagu	2	0	JCK-1
aggcuuaagagugugggug	1	0	LT-1
cuuaagagugugggugguggaaguga	2	0	JT-3
cuuaagagugugggugguggaaguga	2	0	JT-2
cuuaagagugugggugguggaagugauag	2	0	JCK-3
cuuaagagugugggugguggaagugauag	2	0	JCK-2

```
                                                                                                                                     Star                                                                         Mature
cuuuucuuuuuuucaccaaaaucuagacucaauuaauuucucuuuucccuuccuccaccaucugguguauuucuuuucucacuaaaaggauggaagagggaaaggcuuaagagugugggguggaagugauagguaugggggguuaggacagagagaaauacucucuuuuucccaauuuauuuauuuuuuaauugggggaagggaaugaauucuuuccuaccccucccauaccauucauuuccauccuuuc
.........................................................................................................cuuaagagugugggguggaagugauagg.....................................................................................................................    1    0    JCK-3
.........................................................................................................cuuaagagugugggguggaagugauagg.....................................................................................................................    2    0    LT-1
.........................................................................................................cuuaagagugugggguggaagugauagg.....................................................................................................................    1    0    LT-2
.........................................................................................................cuuaagagugugggguggaagugauagg.....................................................................................................................    3    0    JT-3
.........................................................................................................cuuaagagugugggguggaagugauagg.....................................................................................................................    4    0    JT-2
.........................................................................................................cuuaagagugugggguggaagugauagg.....................................................................................................................    5    0    JCK-2
............................................................................................................aagagugugggguggaagugauagg.....................................................................................................................    4    0    JT-3
............................................................................................................aagagugugggguggaagugauagg.....................................................................................................................    2    0    JT-2
................................................................................................................gugugggguggaagugauagg.....................................................................................................................    1    0    JCK-3
................................................................................................................gugugggguggaagugauagg.....................................................................................................................    1    0    JCK-1
.................................................................................................................ugugggguggaagugauaggu....................................................................................................................    10    0    LCK-3
..................................................................................................................gugggguggaagugauagguau..................................................................................................................    1    0    LT-1
..................................................................................................................gugggguggaagugauagguaug.................................................................................................................    8    0    LCK-3
...................................................................................................................ugggguggaagugauagguaugg................................................................................................................    2    0    JCK-3
......................................................................................................................gguggaagugauagguauggg...............................................................................................................    1    0    LT-3
.......................................................................................................................guggaagugauagguaug.................................................................................................................    5    0    LCK-3
.......................................................................................................................guggaagugauagguauggg...............................................................................................................    1    0    LT-1
.......................................................................................................................guggaagugauagguaugggg..............................................................................................................    1    0    JCK-3
.......................................................................................................................guggaagugauagguaugggg..............................................................................................................    2    0    JCK-2
........................................................................................................................uggaagugauagguaugg................................................................................................................    1    0    LT-3
........................................................................................................................uggaagugauagguaugggg..............................................................................................................    2    0    JCK-2
........................................................................................................................uggaagugauagguaugggg..............................................................................................................    1    0    JCK-3
........................................................................................................................uggaagugauagguaugggggg............................................................................................................    6    0    LCK-3
............................................................................................................................agugauagguaugggggguu..........................................................................................................    3    0    LT-3
............................................................................................................................agugauagguaugggggguuaggaca....................................................................................................    7    0    LCK-3
............................................................................................................................agugauagguaugggggguuaggacagaga................................................................................................    2    0    LCK-3
..............................................................................................................................ugauagguaugggggguuaggaca....................................................................................................    3    0    LT-3
................................................................................................................................auagguaugggggguuaggacaga..................................................................................................    1    0    JCK-2
................................................................................................................................auagguaugggggguuaggacaga..................................................................................................    1    0    JCK-1
.................................................................................................................................uagguaugggggguuagga......................................................................................................    4    0    LT-3
.................................................................................................................................uagguaugggggguuagga......................................................................................................    1    0    JCK-1
.................................................................................................................................uagguaugggggguuaggac.....................................................................................................    2    0    JCK-2
.................................................................................................................................uagguaugggggguuaggaca....................................................................................................    6    0    JCK-2
.................................................................................................................................uagguaugggggguuaggaca....................................................................................................    4    0    LCK-3
.................................................................................................................................uagguaugggggguuaggaca....................................................................................................    1    0    LT-3
.................................................................................................................................uagguaugggggguuaggacU....................................................................................................    1    1    LT-3
.................................................................................................................................uagguaugggggguuaggacC....................................................................................................    1    1    LT-3
.................................................................................................................................uagguaugggggguuaggaca....................................................................................................    3    0    LT-1
.................................................................................................................................uagguaugggggAuuaggaca....................................................................................................    1    1    LT-1
.................................................................................................................................uagguaugggggguuaggaca....................................................................................................    3    0    JT-3
.................................................................................................................................uagguaugggggguuaggaca....................................................................................................    1    0    JT-2
.................................................................................................................................uagguaugggggguuaggaca....................................................................................................    1    0    JCK-1
.................................................................................................................................uagguaugggggguuaggacC....................................................................................................    2    1    JCK-3
.................................................................................................................................uagguaugggggguuaggaca....................................................................................................    6    0    JCK-3
.................................................................................................................................uagguaugggggguuaggacaga..................................................................................................    1    0    JCK-3
...................................................................................................................................gguaugggggguuaggacag...................................................................................................    1    0    JCK-3
...................................................................................................................................gguaugggggguuaggacaga..................................................................................................    4    0    JCK-3
...................................................................................................................................gguaugggggguuaggacaga..................................................................................................    4    0    LT-1
...................................................................................................................................gguaugggggguuaggacaga..................................................................................................    3    0    JCK-2
...................................................................................................................................gguaugggggguuaggacaga..................................................................................................    3    0    LT-3
...................................................................................................................................gguaugggggguuaggacaga..................................................................................................    9    0    LT-2
...................................................................................................................................gguaugggggguuaggacaga..................................................................................................    1    0    LCK-3
...................................................................................................................................gguaugggggguuaggacaga..................................................................................................    12    0    JT-1
...................................................................................................................................gguaugggggguuaggacaga..................................................................................................    9    0    JCK-1
....................................................................................................................................guaugggggguuaggacaga..................................................................................................    1    0    LT-3
....................................................................................................................................guaugggggguuaggacaga..................................................................................................    2    0    LT-1
....................................................................................................................................guaugggggguuaggacaga..................................................................................................    6    0    LCK-3
....................................................................................................................................guaugggggguuaggacagag.................................................................................................    1    0    JCK-3
....................................................................................................................................Cuaugggggguuaggacagaga................................................................................................    2    1    JCK-1
....................................................................................................................................Cuaugggggguuaggacagaga................................................................................................    7    1    JT-1
....................................................................................................................................Cuaugggggguuaggacagaga................................................................................................    2    1    JCK-2
....................................................................................................................................Cuaugggggguuaggacagaga................................................................................................    1    1    LT-3
.....................................................................................................................................uaugggggguuaggacag...................................................................................................    18    0    JCK-3
.....................................................................................................................................uaugggggguuCggacag...................................................................................................    1    1    JCK-2
.....................................................................................................................................uaugggggguuaggacag...................................................................................................    8    0    LT-2
.....................................................................................................................................uaugggggguuaggacag...................................................................................................    9    0    JCK-1
.....................................................................................................................................uaugggggguuaggacag...................................................................................................    10    0    LT-3
.....................................................................................................................................uaugggggguuaggacag...................................................................................................    8    0    LT-1
.....................................................................................................................................uaugggggguuaggacag...................................................................................................    14    0    JCK-2
.....................................................................................................................................uaugggggguuaggacag...................................................................................................    10    0    LCK-3
```

Star Mature

cuuucuuuuuuucaccaaaaucuagacucaauuaauuucucuuuucccuuccuccaccaucugguguauuucuuuucucacuaaaaggauggaagagggaaaggcuuaagagugugggugguggaagugauagguaugggggguuaggacagagagaaauacucucuuuuucccaauuuauuuauuuuuuaauuggggggaagggaaugaauucuuuccuaccccucccauaccauucauuuccauccuuuc

uCugggggguuaggacaga	1	1	JCK-1
uaugggggguuaggacaga	11	0	JT-2
uNugggggguuaggacaga	1	1	JT-2
uaugggggguuaggacagU	1	1	LT-1
uaugggggguuaggacaga	14	0	LT-3
uaugggggguuaggacaga	11	0	JT-1
uaugggggguuaggacaga	5	0	JT-3
uaugggggguuaggacaga	12	0	JCK-3
uaugggggguuaggacagU	1	1	JCK-2
uaugggggguuaggacaga	11	0	JCK-2
uaugggggguuaggacaga	19	0	LT-2
uaugggggguuaggacaga	8	0	LT-1
uaugggggguuaggacaga	37	0	LCK-3
uaugggggguuNggacagag	1	1	JCK-1
uaugggggguuaggacagag	35	0	LT-2
uaugggggguuaggacagag	60	0	JCK-1
uaugggggguuaggacagag	16	0	LCK-3
uCugggggguuaggacagag	3	1	JCK-2
uaugggggguuCggacagag	1	1	JCK-2
uaugggggguuaggacagag	20	0	JT-3
uaugggggguuaggacagaN	1	1	JCK-1
uCugggggguuaggacagag	1	1	JT-3
uaugggggguuaggacagag	7	0	JT-2
uaugggggguuaggacagag	34	0	LT-1
uaugggggguuaggacagaU	1	1	LT-2
uaugggggguuaggacagag	62	0	JCK-3
uaugggggguuaggacagag	43	0	LT-3
uaugggggguuaggacagag	115	0	JT-1
uaugggggguuaggacagag	91	0	JCK-2
uaugggggguuaggacagagC	2	1	JCK-1
uaugggggguuaggacagaga	113	0	LCK-1
uCugggggguuaggacagaga	4	1	LT-2
uaugggggguuaggacagaNa	1	1	JCK-1
uaugggggguuaggacagaga	242	0	LT-2
uaugggggguuaggCcagaga	1	1	LCK-1
uaugggggguuaggacagaga	478	0	JCK-1
uaugggggguuagNacagaga	2	1	LCK-1
uaNgggggguuaggacagaga	1	1	LCK-2
uaugggggguuaggacagaga	589	0	JT-1
uCugggggguuaggacagaga	6	1	JCK-2
uaCgggggguuaggacagaga	1	1	JT-3
uaugggggguuCggacagaga	1	1	LCK-3
uauAggggguuaggacagaga	1	1	JCK-2
uaugggggguuaggacagaga	289	0	LT-3
uaugggggguuaggCcagaga	2	1	JCK-2
uaugggggguuaggacagagU	1	1	JCK-1
uCugggggguuaggacagaga	2	1	LCK-3
uaugggggguuaggacagaga	192	0	JT-2
uaugggggguuaggCcagaga	1	1	JT-2
uaugggggguuCggacagaga	1	1	LCK-2
uCugggggguuaggacagaga	1	1	JT-3
uaugggUgguuaggacagaga	1	1	JT-3
uauAggggguuaggacagaga	1	1	JCK-3
uaugggggguuaggacagaga	88	0	LCK-2
uaugggggguuaggCcagaga	1	1	JT-1
uaugggggguuaggacagagU	3	1	LT-3
uaugggggguuaggacagaga	206	0	LT-1
uaugggggguuNggacagaga	1	1	JCK-1
uaugggggguuaggacagaga	727	0	JCK-2
uaugggggguuaggacagCga	1	1	LT-2
uaugggggguuaggCcagaga	1	1	LT-3
uaugggggguuaggacagagU	1	1	LT-1
uaugggggguuaggCcagaga	1	1	JCK-1
uaugggggguuaggCcagaga	1	1	JCK-3
uCugggggguuaggacagaga	3	1	JT-1
uNugggggguuaggacagaga	1	1	JCK-2
uaugggggguuaggacCgaga	1	1	JCK-2
uaugggggguuaggacagaga	231	0	JT-3
uaugggggguuaggacagagC	2	1	JCK-3
uaugggggguuagAacagaga	1	1	LCK-3

Star Mature

cuuuucuuuuuuucaccaaaaucuagacucaauuaauuucucuuuucccuuccuccaccaucugguguauuucuuuucucacuaaaaggauggaagagggaaaggcuuaagagugugggguggaagugauagguaugggggguuaggacagagagaaauacucucuuuuucccaauuuauuuauuuuuuaauuggggggaagggaaugaauucuuuccuaccccucccauaccauucauuuccauccuuuc

uaugggggguuaggacagaga	345	0	LCK-3
uaugggggguuaggacagaga	555	0	JCK-3
uauggggggCuaggacagaga	1	1	JT-2
Caugggggguuaggacagaga	1	1	LT-2
uCugggggguuaggacagaga	1	1	JCK-3
uauAgggggguuaggacagaga	1	1	JT-1
uaugggggguuagUacagaga	1	1	LCK-2
uauAgggggguuaggacagaga	1	1	JCK-1
uauggggggguuCggacagaga	1	1	JCK-3
uaugggggguuaggacagagaU	1	1	JCK-2
uaugggggguuaggacagagaC	1	1	JCK-3
uaugggggguuaggacagagaU	2	1	LT-1
uaugggggguuaggacagagaU	1	1	LT-2
uaugggggguuaggacagagaU	1	1	LT-3
uaugggggguuaggacagagaC	3	1	JCK-2
uaugggggguuaggacagagagaa	2	0	JCK-2
augggggguuaggacaga	1	0	LT-3
augggggguuaggacaga	2	0	LT-2
augggggguuaggacagag	1	0	LT-2
augggggguuaggacagag	3	0	JCK-1
augggggguuaggacagag	1	0	JCK-2
augggggguuaggacagaga	5	0	JCK-3
augggggguuaggacagaga	2	0	JCK-2
augggggguuaggacagagaU	1	1	JCK-2
uggggggguuaggacagaga	3	0	JCK-2
uggggggguuaggacagaga	1	0	JCK-1
ggggguuaggacagagagaaaua	1	0	LT-3
gggguuaggacagagagaaauac	1	0	LT-3
ggguuaggacagagagaaauac	4	0	LCK-3
guuaggacagagagaaauacu	3	0	LCK-3
uuuuuaCuuggggggaaggga	1	1	LCK-3
uuuuuaauuggggggaaggga	5	0	LCK-3
uuaauuggggggaagggaau	1	0	LT-3
uuaauuggggggaagggaaug	1	0	LT-1
uuaauuggggggaCgggaaug	1	1	LT-1
uaauuggggggaagggaaugaau	1	0	LT-3
uaauuggggggaagggaaugaauA	6	1	LCK-3
uaauuggggggaagggaaugaauuc	2	0	LCK-3
uaauuggggggaagggaaugaauuc	2	0	LT-3
aauuggggggaagggaau	6	0	LCK-3
uuggggggaagggaaugaauucuuuc	7	0	LCK-3
uggggggaagggaaugaauucu	1	0	JCK-1
uggggggaagggaaugaauucu	1	0	JCK-2
ggggaagggaaugaauucu	1	0	LT-1
aaugaauucuuuccuaccccuc	7	0	JT-3
aaugaauucuuuccuaccccuc	4	0	JT-2
Cucuuuccuaccccuccca	1	1	LT-1
ucuuuccuaccccuccca	35	0	LCK-2
ucuuuccuaccccuccca	40	0	LT-1
ucuuuccuaccccuccca	9	0	JT-2
ucuuCccuaccccuccca	1	1	LT-2
ucuuuccuaccccuccca	88	0	LT-2
ucuuuccuaccccuccca	16	0	JCK-1
ucuuuccuaccccuccca	33	0	LCK-1
ucuuuccuaccccNccca	1	1	LCK-1
ucuuuccuaccccuccca	67	0	LCK-3
ucuuuccuaccccuccca	22	0	JCK-2
ucuuuccuUccccuccca	3	1	LT-2
ucuuuccuaccccuccca	50	0	LT-3
ucuuuccuaccccucccU	1	1	LCK-3
ucuuuccuaccccuccca	9	0	JT-3
ucuuuccuacAccuccca	1	1	LCK-3
ucuuuccuaccccuccca	3	0	JT-1
ucuuuccuaccccuccca	9	0	JCK-3
ucuuuccuaccccucccau	23	0	LCK-3
ucuuuccuaccccucccau	6	0	JCK-3
ucuuuccuaccccucccau	12	0	JCK-1
ucuuuccuaccccucccau	17	0	LT-1
ucuuuccuaccccucccau	14	0	LT-2
ucuuuccuaccccucccau	25	0	LT-3

Star Mature

```
cuuuucuuuuuuucaccaaaaucuagacucaauuaauuucucuuuucccuuccuccaccaucugguguauuucuuuucucacuaaaaggauggaagagggaaaggcuuaagaguguggguggaagugauagguauggggguuaggacagagagaaauacucucuuuucccaauuuauuuauuuuuuaauugggggaagggaaugaauucuuuccuaccccucccauaccauucauuuccauccuuuc
..............................................................................................................................................................................................................ucuuuccuaccccucccau.....................   26   0   LCK-2
..............................................................................................................................................................................................................ucuuuccuaccccuAccau.....................   1    1   LCK-3
..............................................................................................................................................................................................................ucuuuccuaccccuGccau.....................   1    1   LCK-1
..............................................................................................................................................................................................................ucuuuccuaccccucccau.....................   14   0   JCK-2
..............................................................................................................................................................................................................ucuuuccuaccccucccau.....................   27   0   LCK-1
..............................................................................................................................................................................................................ucuuuccuaccccucccaua....................   59   0   JCK-2
..............................................................................................................................................................................................................ucuuuccuacUccucccaua....................   28   1   JT-1
..............................................................................................................................................................................................................ucuuuccuaccccucccaua....................   41   0   LT-2
..............................................................................................................................................................................................................ucuuuccuaccccucccaua....................   42   0   LT-1
..............................................................................................................................................................................................................ucuuuccuaccccucccaua....................   53   0   LCK-3
..............................................................................................................................................................................................................ucuuuccuaccccucccaAa....................   1    1   JCK-3
..............................................................................................................................................................................................................uNuuuccuaccccucccaua....................   1    1   JCK-2
..............................................................................................................................................................................................................ucuuuccuaccccucccauU....................   2    1   LT-3
..............................................................................................................................................................................................................ucuuuccuaccccucccauU....................   2    1   LT-1
..............................................................................................................................................................................................................ucuuuccuaccccucccauC....................   2    1   LT-3
..............................................................................................................................................................................................................ucuuuccuaccccucccaua....................   43   0   JCK-1
..............................................................................................................................................................................................................ucuuuccuaccccucccaua....................   57   0   JCK-3
..............................................................................................................................................................................................................ucuuuccuaccccucccCua....................   1    1   LT-3
..............................................................................................................................................................................................................ucuuuccuaccccucccaua....................   35   0   LT-3
..............................................................................................................................................................................................................ucuuuccuaccccucccauac...................   7    0   JCK-2
..............................................................................................................................................................................................................ucuuuccuaccccucccauac...................   1    0   JCK-1
..............................................................................................................................................................................................................ucuuuccuaccccucccauac...................   3    0   LT-1
..............................................................................................................................................................................................................ucuuuccuaccccucccauac...................   8    0   LT-2
..............................................................................................................................................................................................................ucuuuccuaccccucccauac...................   4    0   LT-3
..............................................................................................................................................................................................................ucuuuccuaccccucccauaA...................   1    1   LT-1
..............................................................................................................................................................................................................ucuuuccuaccccucccauac...................   5    0   JCK-3
..............................................................................................................................................................................................................ucuuuccuaccccucccauacc..................   26   0   LCK-1
..............................................................................................................................................................................................................uNuuuccuaccccucccauacc..................   4    1   JCK-2
..............................................................................................................................................................................................................ucCuuccuaccccucccauacc..................   1    1   JCK-3
..............................................................................................................................................................................................................ucuuuccuacGccucccauacc..................   2    1   JCK-1
..............................................................................................................................................................................................................uNuuuccuaccccucccauacc..................   1    1   JCK-1
..............................................................................................................................................................................................................ucuuuccuaccccucccauacc..................   48   0   JT-1
..............................................................................................................................................................................................................ucuuucUuaccccucccauacc..................   3    1   JCK-2
..............................................................................................................................................................................................................ucuuuccuacccAucccauacc..................   1    1   LCK-3
..............................................................................................................................................................................................................ucuuuccuaccccucccCuacc..................   6    1   JCK-2
..............................................................................................................................................................................................................ucuuuccuacGccucccauacc..................   2    1   JCK-2
..............................................................................................................................................................................................................ucuuuccuacUccucccauacc..................   80   1   LCK-2
..............................................................................................................................................................................................................uUuuuccuaccccucccauacc..................   3    1   JCK-2
..............................................................................................................................................................................................................ucuuuccuaccccucccauaGc..................   1    1   JCK-1
..............................................................................................................................................................................................................ucuuuccuaccccucccauacc..................   18   0   JT-3
..............................................................................................................................................................................................................ucuuuccuaccccCcccauacc..................   1    1   JCK-2
..............................................................................................................................................................................................................ucuuuccuaccccucccauCcc..................   3    1   JCK-2
..............................................................................................................................................................................................................ucuCuccuaccccucccauacc..................   1    1   LT-3
..............................................................................................................................................................................................................uNuuuccuaccccucccauacc..................   1    1   JCK-3
..............................................................................................................................................................................................................ucuuuccuaccccuccUauacc..................   1    1   JCK-2
..............................................................................................................................................................................................................ucuuuccuaccccucccauCcc..................   1    1   LT-1
..............................................................................................................................................................................................................ucuuuccuaccccucccauCcc..................   1    1   LT-2
..............................................................................................................................................................................................................ucuGuccuaccccucccauacc..................   1    1   JCK-1
..............................................................................................................................................................................................................ucuuuccuaccccuUccauacc..................   4    1   JCK-1
..............................................................................................................................................................................................................ucuuuccuaccccucccauCcc..................   2    1   LT-3
..............................................................................................................................................................................................................ucuuuccuaccccucUcauacc..................   1    1   LCK-3
..............................................................................................................................................................................................................Ccuuuccuaccccucccauacc..................   2    1   LT-2
..............................................................................................................................................................................................................ucuuuccuaccccucccCuacc..................   4    1   JCK-1
..............................................................................................................................................................................................................ucuuuccuaccccuccUauacc..................   2    1   LT-3
..............................................................................................................................................................................................................ucuuuccuacUccucccauacc..................   26   1   JT-3
..............................................................................................................................................................................................................ucuuuccuacUccucccauacc..................   14   1   JT-2
..............................................................................................................................................................................................................Ccuuuccuaccccucccauacc..................   3    1   JCK-2
..............................................................................................................................................................................................................ucuuuccuaccccucccauCcc..................   1    1   JCK-1
..............................................................................................................................................................................................................ucuuuccuaccccucccauaGc..................   1    1   JCK-2
..............................................................................................................................................................................................................ucuuuccuCccccucccauacc..................   1    1   LT-1
..............................................................................................................................................................................................................ucuuuccuaccccucccauacc..................   226  0   LT-2
..............................................................................................................................................................................................................ucuuuccuacGccucccauacc..................   3    1   JCK-3
..............................................................................................................................................................................................................ucuuuUcuaccccucccauacc..................   1    1   JCK-1
..............................................................................................................................................................................................................ucuuuccuaccccucccauacc..................   214  0   LCK-3
..............................................................................................................................................................................................................ucuuuccuaccccucccauacA..................   1    1   JCK-2
..............................................................................................................................................................................................................ucCuuccuaccccucccauacc..................   1    1   LT-2
..............................................................................................................................................................................................................ucuuCccuaccccucccauacc..................   1    1   JCK-3
..............................................................................................................................................................................................................ucuuuccuacUccucccauacc..................   87   1   LCK-1
..............................................................................................................................................................................................................ucuuuccuCccccucccauacc..................   7    1   JCK-2
..............................................................................................................................................................................................................uUuuuccuaccccucccauacc..................   1    1   JCK-1
```

Star: uauggggguuaggacagaga — Mature: ucuuuccuaccccucccauacc

cuuuucuuuuuuucaccaaaaucuagacucaauuaauuucucuuuucccuuccuccaccaucugguguauuucuuuucucacuaaaaggauggaagagggaaaggcuuaagagugugggguggaagugauagguauggggguuaggacagagagaaauacucucuuuuucccaauuuauuuauuuuuuaauugggggaagggaaugaauucuuuccuaccccucccauaccauucauuuccauccuuuc

ucuuuccuCcccucccauacc	3	1	JCK-3
ucuuuccuaccccucccCuacc	2	1	JCK-3
ucuuucUuaccccucccauacc	1	1	JCK-1
ucuuuccuaccccucccGuacc	1	1	JCK-1
ucuuuccuaccNcucccauacc	3	1	JCK-1
ucuuuccuacUccucccauacc	1	1	JCK-2
ucuuuccuaccccucccauacU	1	1	LT-1
ucuuuccuacUccucccauacc	114	1	JT-1
ucuuuccuaccccuUccauacc	1	1	JCK-3
ucuuuccuaccccucccauNcc	1	1	JCK-1
Ccuuuccuaccccucccauacc	1	1	JCK-3
ucuuuccuCcccucccauacc	2	1	LT-3
uUuuuccuaccccucccauacc	1	1	JCK-3
ucuuuccuaccccucccauacc	419	0	LT-3
ucuuuccuaccccucccauaUc	1	1	JCK-3
ucCuuccuaccccucccauacc	1	1	JCK-2
ucuuuUcuaccccucccauacc	4	1	JCK-2
ucuuuccuaccccucccauacc	18	0	LCK-2
ucuuuccuaccccucccCuacc	1	1	LT-2
ucuuuccuaccccucccauacc	1146	0	JCK-1
ucuuuccuaccccucccauacc	1673	0	JCK-3
ucuuuccuaUcccucccauacc	3	1	LT-3
ucuuuccuaccccucccauacc	434	0	LT-1
ucuuuccuaccccucccauaUc	1	1	JCK-2
ucuuuccuaAcccucccauacc	2	1	JCK-1
ucuuuccuaccccucccauacc	2190	0	JCK-2
ucuuuccuaccccucccauacc	29	0	JT-2
ucuuuccuaccccucccauacU	2	1	JCK-1
ucCuuccuaccccucccauacc	1	1	JCK-1
ucuuuccuaccccucccauaccU	6	1	JCK-1
ucuuuccuaccccucccauacca	2	0	LT-3
ucuuuccuaccccucccauaccU	2	1	JCK-2
ucuuuccuaccccucccauaccU	2	1	JCK-3
ucuuuccuaccccucccauacca	1	0	LT-1
ucuuuccuaccccucccauacca	1	0	JCK-2
ucuuuccuaccccucccauacca	1	0	JCK-1
ucuuuccuaccccucccauaccU	5	1	LT-3
ucuuuccuaccccucccauaccU	11	1	LT-1
ucuuuccuaccccucccauaccUu	2	1	LT-2
ucuuuccuaccccucccauaccaA	3	1	LT-2
ucuuuccuaccccucccauaccUu	6	1	LCK-3
ucuuuccuaccccucccauaccUuu	1	1	LT-3
ucuuuccuaccccucccauaccUuu	3	1	LT-2
ucuuuccuaccccucccauaccaCuc	1	1	JCK-2
ucuuuccuaccccucccauaccauuG	1	1	LT-1
cuuuccuaccccucccau	1	0	JCK-3
cuuuccuaccccucccau	2	0	LCK-3
cuuuccuaccccucccaua	1	0	JCK-3
cuuuccuaccccucccaua	1	0	JCK-1
cuuuccuaccccucccaua	4	0	LCK-3
cuuuccuaccccucccauacc	16	0	JCK-3
cuuuccuaccccucccauacc	6	0	LT-3
cuuuccuaccccucccauacc	11	0	JCK-2
cuuuccuaccccucccauacc	11	0	JCK-1
cuuuccuaccccucccauaccauuU	2	1	JCK-3
cuuuccuaccccucccauaccauuU	2	1	JCK-2
uuuccuaccccucccaua	1	0	JCK-2
uuuccuaccccucccaua	4	0	LCK-3
uuuccuaccccucccaua	1	0	LT-2
uuuccuaccccucccaua	5	0	JCK-3
uuuccuaccccucccaua	2	0	LT-3
uuuccuaccccucccaua	1	0	LT-1
uuuccuaccccucccauU	1	1	LT-3
uuuccuaccccucccauacc	8	0	LT-2
uuuccuCcccucccauacc	2	1	LT-3
Cuuccuaccccucccauacc	2	1	LT-2
uuuccuaccccucccauacU	1	1	LT-1
uuuccuaccccucccauacc	18	0	JCK-2
uuuccuaccccucccauacc	33	0	LT-1
uuuccuaccccucccauacc	11	0	LCK-3

Star **Mature**

cuuucuuuuuuucaccaaaaucuagacucaauuaauuucucuuucccuuccuccaccaucugguguauuucuuuucucacuaaaaggauggaagagggaaaggcuuaagagugugggguggaagugauagguaugggggguuaggacagagagaaauacucucuuuucccaauuuauuuauuuuuuaauuggggggaagggaaugaauucuuuccuaccccucccauaccauucauuuccauccuuuc

…uuuccuaccccucccauacc…	28	0	LT-3
…uuuccuaccccucccauacc…	10	0	JCK-3
…uuuccuaccccucccauacc…	12	0	JCK-1
…uuuccuaccccucccauaccU…	1	1	JCK-3
…uuuccuaccccucccauacca…	1	0	JCK-2
…uuuccuaccccucccauacca…	1	0	LT-3
…uuuccuaccccucccauacca…	2	0	LT-1
…uuuccuaccccucccauacca…	2	0	JCK-3
…uuuccuaccccucccauacca…	1	0	JCK-1
…uuuccuaccccucccauaccU…	2	1	LT-1
…uuuccuaccccucccauacca…	3	0	LCK-3
…uuuccuaccccucccauaccU…	1	1	JCK-1
…uuuccuaccccucccauaccU…	1	1	JCK-2
…uuuccuaccccucccauacca…	2	0	JT-1
…uuuccuaccccucccauaccUu…	1	1	LT-1
…uuuccuaccccucccauaccau…	10	0	JCK-1
…uuuccuacccAucccauaccau…	1	1	JCK-2
…uuuccuaccccucccauaccau…	7	0	LT-3
…uNuccuaccccucccauaccau…	1	1	JCK-2
…uuuccuaccccucccauaccau…	4	0	LT-1
…uuuccuaccccucccauaccau…	7	0	JCK-3
…uuuccuaccccucccauaccau…	5	0	JT-2
…uuuccuaccccucccauaccau…	2	0	LCK-3
…uuuccuaccccucccauaccau…	15	0	JCK-2
…uuuccuaccccucccauaccau…	6	0	JT-3
…uuuccuaccccucccauaccauu…	3	0	LT-3
…uuuccuaccccucccauaccauu…	3	0	LT-1
…uuuccuaccccucccauaccauu…	3	0	JCK-2
…uuuccuaccccucccauaccUuu…	2	1	LT-2
…uuuccuaccccNcccauaccauu…	1	1	JCK-1
…uuuccuaccccucccauaccauu…	2	0	LT-2
…uuuccuacccGucccauaccauu…	1	1	LT-2
…uuuccuaccccucccauaccauu…	2	0	JCK-3
…uuuccuaccccucccauaccauuc…	1	0	LT-3
…uuuccuaccccucccauaccauuc…	1	0	LCK-3
…uuuccuaccccucccauaccauuc…	3	0	JCK-2
…uuuccuaccccucccauaccauuc…	1	0	JCK-1
…uuuccuaccccucccauaccauuU…	1	1	LT-1
…uuuccuaccccucccauaccauuc…	3	0	LT-1
…uuuccuaccccucccauaccauuc…	6	0	JCK-3
…uuccuaccccucccauacc…	8	0	JT-1
…uuccuaccccucccauacc…	6	0	LCK-3
…uuccuaccccucccauacc…	1	0	LT-3
…uuccuaccccucccauacc…	3	0	LT-1
…uuccuaccccucccauaccauu…	2	0	LT-1
…uuccuaccccucccauaccauu…	2	0	JCK-3
…uAccuaccccucccauaccauu…	2	1	JCK-3
…uAccuaccccucccauaccauu…	2	1	JCK-1
…uuccuaccccucccauaccauu…	1	0	JCK-2
…uuccuaccccucccauaccauu…	5	0	LCK-3
…uuccuaccccucccauaccauuU…	1	1	LT-1
…uuccuaccccucccauaUcauuc…	1	1	JCK-2
…uuccuaccccucccauaccauuc…	11	0	JCK-2
…uuccuaccccucccauaccauuc…	4	0	JCK-1
…uuccuaccccucccauaccauuc…	4	0	JCK-3
…uccuaccccucccauacc…	1	0	LT-2
…uccuaccccucccauacc…	2	0	LT-3
…uccuaccccucccauacc…	5	0	JT-3
…uccuaccccucccauacc…	1	0	JCK-2
…uccuaccccucccauacc…	3	0	JT-2
…uccuaccccucccauacc…	2	0	LCK-3
…uccuaccccucccauacc…	1	0	LT-1
…uccuaccccucccauacc…	1	0	JCK-1
…uccuaccccucccauacca…	5	0	JT-3
…uccuaccccucccauacca…	5	0	LT-2
…uNcuaccccucccauaccauu…	1	1	JCK-3
…uccuaccccucccauaccauu…	11	0	LT-3
…uccuaccccucccauaccauu…	53	0	JCK-3
…uccuaccccucccauaccauu…	5	0	LT-2
…uccuaccccucccauaccauu…	20	0	LT-1

Star | Mature

cuuucuuuuuuucaccaaaaucuagacucaauuaauuucucuuuucccuuccuccaccaucugguguauuucuuuucucacuaaaaggauggaagagggaaaggcuuaagagugugggugga agugauagg**uaugggggguuaggacagaga**gaaauacucucuuuuucccaauuuauuuauuuuuuaauuggggggaagggaaugaau**ucuuuccuaccccucccauacc**auucauuuccauccuuuc

uccuacccAucccauaccauu	1	1	JCK-2
uccuaccccucccaCaccauu	1	1	JCK-2
uccuaccccucccauaccauu	85	0	JCK-2
uccuaccccucccauaccCuu	1	1	JCK-1
uccuaccccucccauaccauu	52	0	JCK-1
uccuaccccucccauaccauuc	5	0	LT-2
uccuaccccucccauaccauuU	3	1	JCK-1
uccuaccccucccauaccauuU	5	1	JT-3
uccuaccccucccauaccauuc	66	0	JCK-1
uccuaccccuAccauaccauuc	1	1	JCK-1
uccuCccccucccauaccauuc	1	1	JCK-2
Cccuaccccucccauaccauuc	1	1	JCK-2
uccuaccccucccauaccauuc	101	0	JCK-3
uccuaccccucccauaccauuc	16	0	LT-1
uccuaccccucccauaccauuU	1	1	LT-1
uccuaccccucccauaccauuA	1	1	JCK-3
uccuaccccucccauaccauuc	23	0	LT-3
uccuacccAucccauaccauuc	1	1	JCK-3
uccuaccccucccauaccauuU	3	1	JCK-3
uccuaccccucccauaccauuU	2	1	LT-3
uccuaccccucccauaccauuU	6	1	JCK-2
uccuaccccucccauCccauuc	1	1	JCK-1
uccuaccccucccCuaccauuc	1	1	JCK-2
uccuaccccucccauaccauuc	142	0	JCK-2
uccuaccccucccauaccauuU	3	1	JT-2
uccuaccccucccauaccauuc	8	0	LCK-3
ccuaccccucccauacca	1	0	LT-3
ccuaccccucccauacca	2	0	LT-2
ccuaccccucccauaccau	3	0	JCK-3
cUuaccccucccauaccauu	1	1	JCK-2
ccuaccccucccauaccauu	12	0	JCK-3
ccuaccccucccauaccauu	4	0	LT-2
ccuaccccucccauaccauu	3	0	LT-3
ccuaccccucccauaccauu	3	0	LT-1
ccuaccccucccauaccauu	5	0	JCK-1
ccuaccccucccauaccauu	1	0	LCK-3
ccuaccccucccauaccauu	20	0	JCK-2
ccuaccccucccauaccauuc	22	0	JCK-1
ccuaccccucccauaccauuU	2	1	JCK-1
ccuaAcccucccauaccauuc	1	1	JCK-1
ccuaccccucccauaccauuU	5	1	JCK-2
ccuaccccucccauaccauuU	1	1	LT-3
ccuaccccucccauaccauuc	5	0	LT-3
ccuaccccCcccauaccauuc	1	1	JCK-2
ccuaccccucccauaccauuc	34	0	JCK-2
ccuaccccucccauaccauuc	4	0	LT-1
ccuaccccucccauaccauuU	5	1	JCK-3
ccuaccccucccauaccauuc	32	0	JCK-3
ccuaccccucccauaccauuca	1	0	JCK-1
ccuaccccucccauaccauucU	3	1	LT-2
cuaccccucccauaccau	3	0	LT-1
cuaccccucccauaccaCu	1	1	LT-2
cuaccccucccauaccauu	3	0	JCK-2
cuaccccucccauaccauu	5	0	LT-3
cuaccccucccauaccauu	4	0	LCK-3
cuaccccucccauaccauu	6	0	LT-1
Uuaccccucccauaccauu	1	1	LT-1
cuaccccucccauaccauu	1	0	LT-2
cuaccccucccauaccauuc	6	0	LT-3
cuaccccucccauaccauuc	5	0	JCK-2
cuaccccucccauaccauuc	3	0	JCK-3
cuaccccucccauaccauuU	2	1	JCK-3
cuaccccucccauaccauuU	1	1	JCK-2
cuaccccucccauaccauuc	5	0	LT-2
cuCcccucccauaccauuc	1	1	LT-3
cuaNcccucccauaccauuc	1	1	JCK-1
cuaccccucccauaccauuU	1	1	LT-2
cuaccccucccauaccauuc	3	0	JCK-1
cuaccccucccauaccauuU	3	1	LCK-3
cuaccccucccauaccauuc	4	0	LT-1

```
                                                                                                                                               Star                                                    Mature
cuuucuuuuuuucaccaaaaucuagacucaauuaauuucucuuuucccuuccuccaccaucugguguauuucuuuucucacuaaaaggauggaagagggaaaggcuuaagaguguggguggaagugauagguaugggggguuaggacagagagaaauacucucuuuuucccaauuuauuuauuuuuuaauugggggaagggaaugaauucuuuccuaccccucccauaccauucauuuccauccuuuc
......................................................................................................................................................................................................................cuaccccucccauaccauuU..............   1   1   LT-1
......................................................................................................................................................................................................................cuaccccucccauaccauuca.............   1   0   LT-1
......................................................................................................................................................................................................................cuaccccucccauaccauuca.............   2   0   JCK-1
......................................................................................................................................................................................................................cuaccccucccauaccauucU.............   1   1   LCK-3
.......................................................................................................................................................................................................................uaccccucccauaccauuc..............   1   0   LT-1
...............................................................................................................................................................................................................................ccauaccauucauuuccauccu...   2   0   LT-1
...............................................................................................................................................................................................................................ccauaccauucauuuccauccuAu.   1   1   JCK-3
...............................................................................................................................................................................................................................ccauaccauucauuuccauccuAu.   1   1   JCK-2
................................................................................................................................................................................................................................cauaccauucauuuccauU.....   1   1   JCK-2
................................................................................................................................................................................................................................cauaccauucauuuccauc.....   1   0   LT-1
................................................................................................................................................................................................................................cauaccauucauuuccauccu...   1   0   LT-3
................................................................................................................................................................................................................................cauaccauucauuuccauccuu..   1   0   JCK-2
................................................................................................................................................................................................................................cauaccauucauuuccauccuu..   2   0   JCK-3
................................................................................................................................................................................................................................cauaccauucauuuccauccuuu.   1   0   JCK-2
................................................................................................................................................................................................................................cauaccauucauuuccauccuuu.   2   0   JCK-3
................................................................................................................................................................................................................................cauaccauucauuuccauccuuuc   2   0   JCK-1
.................................................................................................................................................................................................................................auaccauucauuuccauc.....   1   0   LT-3
.................................................................................................................................................................................................................................auaccauucauuuccauccuu..   1   0   LT-3
.................................................................................................................................................................................................................................auaccauucauuuccauccuuu.   1   0   LT-3
.................................................................................................................................................................................................................................auaccauucauuuccauccuuuc   1   0   LT-2
.................................................................................................................................................................................................................................auaccauucauuuccauccuuuU   1   1   JCK-3
.................................................................................................................................................................................................................................auaccauucauuuccauccuuuc   1   0   LT-1
```
